# MPHASYS: a mouse phenotype analysis system

**DOI:** 10.1186/1471-2105-8-183

**Published:** 2007-06-06

**Authors:** R Brent Calder, Rudolf B Beems, Harry van Steeg, I Saira Mian, Paul HM Lohman, Jan Vijg

**Affiliations:** 1 Buck Institute for Age Research, Novato, California, USA; 2 National Institute for Public Health and the Environment, Bilthoven, The Netherlands; 3 Lawrence Berkeley National Laboratory, Berkeley, California, USA

## Abstract

**Background:**

Systematic, high-throughput studies of mouse phenotypes have been hampered by the inability to analyze individual animal data from a multitude of sources in an integrated manner. Studies generally make comparisons at the level of genotype or treatment thereby excluding associations that may be subtle or involve compound phenotypes. Additionally, the lack of integrated, standardized ontologies and methodologies for data exchange has inhibited scientific collaboration and discovery.

**Results:**

Here we introduce a Mouse Phenotype Analysis System (MPHASYS), a platform for integrating data generated by studies of mouse models of human biology and disease such as aging and cancer. This computational platform is designed to provide a standardized methodology for working with animal data; a framework for data entry, analysis and sharing; and ontologies and methodologies for ensuring accurate data capture. We describe the tools that currently comprise MPHASYS, primarily ones related to mouse pathology, and outline its use in a study of individual animal-specific patterns of multiple pathology in mice harboring a specific germline mutation in the DNA repair and transcription-specific gene Xpd.

**Conclusion:**

MPHASYS is a system for analyzing multiple data types from individual animals. It provides a framework for developing data analysis applications, and tools for collecting and distributing high-quality data. The software is platform independent and freely available under an open-source license [1].

## Background

As the volume, complexity and breadth of biological data collected on model organisms increases, more flexible and extensible systems for data collection, integration and analysis are required. Existing systems often focus on data obtained in a specific context. For example, the Knockout Mouse Project [2] aims to generate mouse embryonic stem cells containing a null mutation in every gene in the mouse genome; the Mouse Phenome Project [3] and the Mouse Genome Database [4] aim to gather and disseminate baseline phenotypic data for a defined set of inbred mouse strains; the Mouse Tumor Biology Database [5] maintains information on the mouse as a model system of hereditary cancer; and Pathbase [6] is a database of histopathology images derived from mutant or genetically manipulated mice annotated using a systematized ontology (MPATH). Although these systems expand our understanding of particular phenotypes, they focus on experimental observations associated with classes of animals rather than multiple types of data linked to individual animals, thereby prohibiting researchers from integrating diverse data collected by their own laboratories or others on *individual *animals.

The preliminary hurdle for working with animal data is its collection. Open-source applications like *MouseTRACS *[7] provide mechanisms for mouse colony and protocol management with some facilities for the collection of animal phenotype data. *MuTrack *[8] also provides mechanisms for animal management and data collection in a collaborative context. *MUSDB *[9] is a communications platform based on multiple applications for management of husbandry, mating, ENU injection, sample management, and phenotypic screens. These centralized resources provide mechanisms for collection of basic phenotype data for comparison among animals. However, these data collection systems are husbandry oriented and do not address integration and analysis of histopathological data or "omics" data.

Systems that describe data with complex relationships require the use of expert-curated ontologies for interoperability and precision [10]. The Open Biomedical Ontologies (OBO) initiative is a collection of orthogonal ontologies and includes the Adult Mouse Anatomical Dictionary [11] and the MPATH Mouse Pathology Ontology [6]. The Mammalian Phenotype (MP) ontology [12] annotates "mammalian phenotypes in the context of mutations, quantitative trait loci and strains that are used as models of human biology and disease". These formalizations of pathology and anatomy terms represent community involvement in the development of standardized methodologies for the description of where (anatomy) and what (pathology) in a computer readable format. The individual ontologies are orthogonal and do not typically define relationships between terminologies.

Once primary data is collected, the capacity to combine disparate data sets is a critical component of the ability to generate biological hypotheses [13,14]. In order to facilitate integrated phenotypic analysis of normal, genetically modified or environmentally perturbed mice at the level of single animals, we developed a computational framework for data collection, management and integrated discovery that we call the Mouse Phenotype Analysis System (MPHASYS) [1].

The three main features of MPHASYS are 1) a centralized framework for the collection and analysis of animal data, 2) applications for management, analysis and visualization of animal data using this framework and 3) specifications for standardized collection (predefined phenotypic variables) and dissemination of animal data through an open and extensible data document format. As a framework, MPHASYS unifies and utilizes disparate data related to clinical, pathological and molecular variables obtained from individual animals within the context of existing data sources such as NCBI Gene [15] and the Gene Ontology (GO) [16]. In MPHASYS, the individual animal constitutes the fundamental biological unit to which all data are related. This leads to an animal in a study being a "phenome", *i.e.*, a collection of the measured phenotypic variables that describe the animal. An animal-centric schema defines the relationships of the data and links them to relational databases. The data entry application built upon this framework is a tool designed to provide high quality data collection at the bench with simultaneous access to tools for analysis of the data. The entry of data are guided by means of an interrelated set of ontologies and users are required to enter a minimally defined set of terms for each phenotypic variable. The application provides for the capture of high quality data by requiring that data is complete, matches the format of the data in the database, and prevents inadvertent modification of existing records. This report describes the implementation of the MPHASYS system and presents a case-study using data collected from mice harboring a specific germline mutation in the DNA repair and transcription-specific gene Xpd. This mouse model mimics the human mutation which gives rise to trichothiodystrophy (Xpd^*TTD*^).

## Implementation

The MPHASYS system was written in the Java programming language. An animal-centric schema was designed based on animal data relationships and Hibernate was employed to map data and relationships to relational databases (*e.g. *PostgreSQL [17] or HSQLDB [18]). Existing data such as NCBI Gene were mapped using the data relationships defined by its creators. Mapped data sources were linked using the Spring Framework [19] thereby allowing multiple Hibernate-mediated data sources to be compared. Internally, the MPHASYS framework allows comparison and selection across databases using namespace/identifier pairs which are represented by unique LSID [20] identifiers, allowing data from different databases to be joined via these unique individual animal identifiers. Web based applications were written in Java and run on the JBoss [21] platform. The MPHASYS data entry application was written in Java and uses Java Web-Start for distribution.

The Protégé ontology editor and knowledge-base framework [22] was utilized for development and ontology management. The MPHASYS pathology ontology is based on the National Toxicology Project (NTP) Pathology Code Tables (PCT) from the NIEHS [23]; MPATH pathological instance codes [6]; studies of aging phenotypes in wild-type and DNA-repair deficient mice [24]; standardized reference texts [25,26]; and interaction with rodent pathologists. Terms are entered into Protégé utilizing property slots that encode relationships between terms, organs and scores (see results).

Animal and pathology data were entered into MPHASYS using the Java data entry tool and standardized XML document formats designed for loading data from files. Data, applications and detailed descriptions of the systems architecture and pathology ontology are available online [1].

## Results and discussion

### Design of system architecture

**Figure 1:**
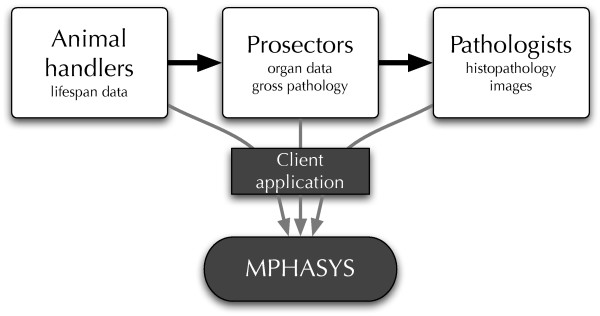
**MPHASYS workflow**. Animal handlers manage the day-to-day collection of clinical variables for individual animals. Prosectors perform analysis of gross pathology and generate samples and slides. Pathologists collect micropathology data and enter it into the system. Investigators utilize MPHASYS to analyze animal data and compare individuals and groups.

**Figure 2:**
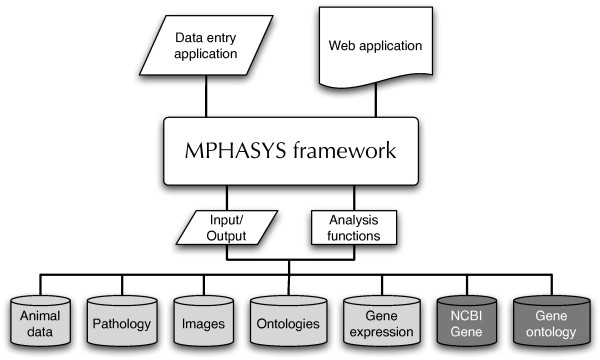
**MPHASYS system architecture**. The MPHASYS framework can operate in either client or server mode, according to the user's requirements. Applications for data collection and analysis utilize the framework to access either integral (light color) or integrated databases (dark color) and to perform analyses. Documents can be imported and exported via the *MouseML *document format for storage or sharing between laboratories.

The architecture of the system is based around the activities of scientists at the bench in order to maximize the probability of accurately capturing data. Figure 1 depicts the workflow of investigators at each stage of data collection and Figure 2 depicts the architecture of the system. MPHASYS is designed to be flexible in how its use and its databases are configured, allowing applications to be written to meet the needs of different data collection and analysis schemes. MPHASYS-based applications can operate independently of each other and data merged via a common document format, they can operate simultaneously using the same database, or external data can be translated to a common document format and imported into the system.

### Modeling animal data and XML schemata

**Figure 3:**
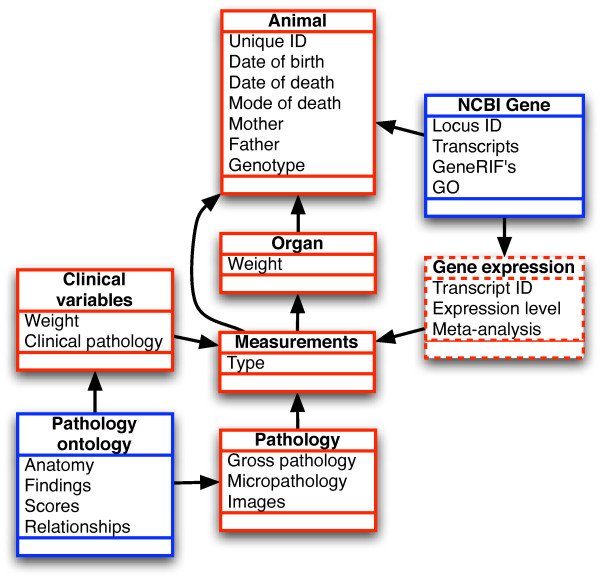
**Mapping of animal data types to individual animals**. Individual animals are the base entity in the mapping, with each animal having collections of organs to which pathologies or other measurements (*e.g. *gene expression data) can be assigned. Items in blue represent extant meta-data which is mapped into the system, dotted boxes represent an example of other databases that can be included in the system.

The heart of MPHASYS is its object-relational mapping of animal measurements to individual animals. Figure 3 shows the primary classes representing the animal data that are persisted within the system. The "Animal" class represents the primary meta-data for individual animals and includes date of birth, date and circumstance of death, unique identifiers and identifiers representing the animal's parents. The animal model specifies the pieces of information collected about an animal, their relationships and the context in which they are to be analyzed. "Organs" are modeled as a many-to-one part-of relationship with the animal.

Organs as well as animals can have "Measurements" which are pre-defined classes of measurement types (*e.g. *body weight in grams) or individually defined ontology-mediated measurement types (*e.g. *clinical variables and pathologies). Organs can be assigned other measurement types (*e.g. *gene expression data).

Unique identifiers are central to maintaining data integrity within the system. MPHASYS utilizes the Life Science Identifiers (LSID) [20] universal resource name (URN) style for describing unique entities. All entities which might be interchangeable (ontologies) or non-unique between systems (animal, study, genotype identifiers) utilize a LSID for its unique identifier. Animal identifiers represent a slightly different usage of the LSID URN format, as each lab is its own naming authority. Therefore, individual laboratories define their own namespaces to keep animal identifiers unique (see supplementary materials online).

A standardized document format was designed using XML Schema [27]. This document format, termed *MouseML *[28], provides a methodology for saving, exchanging, and generating animal data, which validates that the data is complete and properly formatted. *MouseML *replicates the definitions defined by the animal model and defines a polymorphic measurement type, allowing for later expansion of measurement types and the addition of new ontologies or the use of alternate ontologies. MPHASYS is able to read and write animal data documents that satisfy the constraints of the schema. When loading documents, they are automatically evaluated by the system against the schema, ensuring the integrity of the document's structure. Documents conforming to the *MouseML *schema extend the notion of identifying entities via a unique identifier to allow for safe exchange of data across laboratories. The *MouseML *schema definition files and additional information are available online [28].

### MPHASYS ontology

#### General considerations

**Figure 4:**
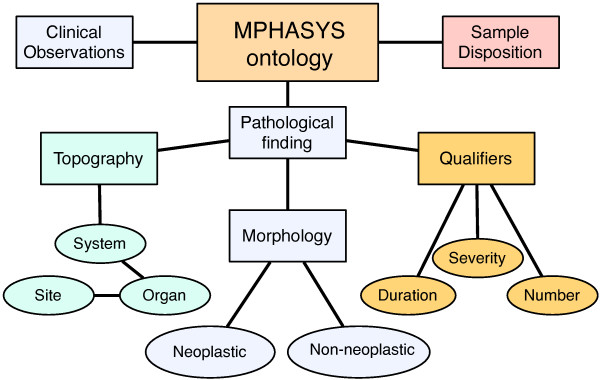
**Organization of the MPHASYS ontology**. The ontology is used to describe pathological findings for clinical observations, gross- and micro-pathology. Clinical observations represent findings made over the life-span of an animal; sample dispositions are a class of terms used to describe why observations can not be made. Pathological findings are tripartite in form, with classes of terms for describing the topography (physical location), morphology (the finding) and qualifiers (morphology specific scores; *e.g. *duration, number and severity).

The emphasis on clinico-pathological phenotypes stems from the importance of multiple pathology as the main criterion for judging the possible effects of germline genotypic changes and/or environmental exposures. While genome-scale, molecular data sets are important, pathophysiological alterations remain the primary basis for characterizing the phenotypes of mouse models. To address this need, we generated a structured mouse-specific pathology ontology based on the NTP PCT [23]. This terminology describes topography (anatomical location: system, organ, site and locative qualifiers), morphologies (pathological findings) and qualifiers (pathology-specific scores: Figure 4) as well as the morphology-topography and morphology-qualifier relationships. Our interest in the histopathological analysis of DNA repair-deficient mutant mice and studies of exposure of these models to DNA damaging agents makes the NTP PCT well-suited as a basis for our terminology: the NTP PCT represent terms developed for rodent exposure studies and are unique in establishing linkages between rodent anatomy and pathology, and over 800 studies of rodent exposure studies performed using these terms are available for download [29].

In addition to the core terms and relationships adopted from the NTP terminology, the lexicon was broadened by the addition of age-related phenotype terms. These terms were collected from several complete life-span studies of wild-type and mutant mice harboring knock-out mutations and mutational mimics of human progeroid syndromes in DNA repair related proteins (*e.g. *[24]). These terms were normalized to remove duplicated terms (alternate terms and spellings) and separate topography-morphology term pairs. The MPHASYS ontology was then expanded to include terms from existing terminologies (MPATH and MA). As the MPHASYS ontology is a synthesis of multiple terminology types, it can also provide relationships between existing terminologies; *i.e. *the ontology links morphologies to topographies and qualifiers using property slots that represent "malignancy type", "micro/gross type", "morphology-organ link", "morphology-qualifier link", "site-organ link", "sex-specificity", and slots for MA, MPATH, and NTP codes. Finally, because of the modular nature of MPHASYS, and its use of unique identifiers at every level, it would also be possible to replace the current MPHASYS ontology with another if required.

#### Topography terminology

The topography component of the MPHASYS ontology describes locations of findings. The terminology is organ based, organizing organs into a PartOf hierarchy that defines the animal as the root of a directed acyclic graph, and organs are classified into classes of organ systems. Associations of anatomical sub-structures (sites) are defined per organ, and organs are labeled with sex specificity. Additionally, locative qualifiers are defined to give more specific detail to description of location within sites. Organs have been annotated with their corresponding Mouse Anatomical Dictionary (MA) organ codes.

#### Pathology terminology

The pathology (morphology) terminology is an IsA hierarchy of terms divided into three primary classes: "neoplastic", "non-neoplastic", and "not remarkable". All terms are linked to the organs for which they are appropriate to describe, allowing data entry and analysis systems to provide a level of data validation. Each term has a link to appropriate types of scores (qualifiers) for each term. Terms are classified as either appropriate for gross- or micropathological examination. Additionally, neoplastic terms are described as malignant and/or benign. Finally, the MPHASYS ontology was updated to completely subsume terms in the MPATH ontology, and existing terms have been annotated with MPATH codes. The pathology terminology currently consists of 787 unique terms.

#### Scores, measurements and clinical observations

Terms directly defined by the ontology include qualifiers, or morphology specific scores, and clinical observations. Qualifiers represent quantitative measures of number, differentiation stage, distribution type, duration, level of inflammation, and severity. Clinical observations describe gross and behavioral findings of animals over the course of their life span, as observed by animal handlers. Classes of clinical observations are behavior, general (basic physical characteristics), clinical lesion, site of application (for describing treatment with chemicals), and cause of death.

The MPHASYS application itself defines a measurement class and several measurement types as well as definitions of units appropriate for recording them. These include weight and lifespan.

### Software engineering and framework implementation

Based upon the animal data model and ontology specifications, a framework was designed to manage and analyze data. We utilized the Hibernate object-relational mapping tool [30] to create an animal-centric schema (Figure 3) which defines data relationships and maps them to a relational database, programmatically representing the animal data and relationships as objects and persisting them in the underlying database. In addition to the core animal data, MPHASYS utilizes data from external sources (NCBI Gene, GeneRIF, Gene Ontology) as ontologies to describe genotypes, molecular data and functional relationships. These external databases were mapped using Hibernate to model the relationships defined by the developers of the data. MPHASYS was designed to provide a mechanism for updating these databases from public repositories.

These mapped data sources were linked using the Spring Framework [19] thereby allowing multiple Hibernate-mediated data sources to be compared. Each sub-system within MPHASYS is configured such that new systems can be integrated easily or existing ones can be replaced (see online documentation).

Using unique identifiers, MPHASYS links data from the disparate databases integrated within. Functions were written that provide a mechanism for selecting and partitioning animals based on user-defined criteria. First, the user selects the animals to be analyzed based on a definition of which studies, genotypes, sex and measurements they wish to incorporate (selection criteria). Second, these animals are partitioned into different comparison groups (series criteria). By default, these groups are generated by dividing animals first by study, then by genotype and finally by sex. However, multiple criteria can also be defined such as divide by presence or absence of a specific pathology and these criteria can be grouped using logical "and" and "or" clauses. The system automatically selects animals based on the selection criteria and performs statistical tests and graphical representation based on divisions defined by the series criteria. MPHASYS provides for unit conversion, annotation and comparison of pathology data utilizing the MPHASYS ontology and visualization and analysis of animal data using the defined selection criteria. Finally, the framework provides analysis tools that are accessible through a web-server.

#### Data entry client application

In order to facilitate accurate collection of animal data, a data entry application was written which utilized the framework. The data entry application (MPHASYS client [31]), was written in Java and utilizes Java Web-Start for distribution. The client application was designed around the animal model and the following steps in data collection: 1) study design and genotype definition, 2) entry of individual animals and generation of cage cards, 3) collection of life-span data (animal weight, clinical observations), 4) collection and gross examination of organs by a prosector and 5) histopathological analysis of organ sections.

Forms were designed for entering study protocol, meta-data and genotypes. The system relies upon NCBI Gene as the basis for annotating genetic interventions and the database tracks an animal's allelic make-up. Forms for annotating individual animals allow the entry of date of birth, sex, genotype, parental data and identifiers (which may be generated automatically). Once animals are entered into the system, cage cards may be generated that contain information about the animal, including identifying meta-data (ear-punch) and a barcode that can be used to quickly select the animal using the client. Once an animal is selected, the application presents the user with forms for management and entry of life-span data, organ data (prosector-generated) and histopathology. Forms for entering life-span data (*e.g *weight) minimize keystrokes and are compatible with automated data entry equipment (balance-keyboard interfaces). Forms for clinical observations, gross- and micro-pathology query the framework for relevant terms based on rules defined by the MPHASYS ontology. Keystrokes can be used to quickly subset terms (the system scores and ranks terms by letters typed by the user) and navigate through menus to select appropriate terms. The system displays only morphological terms and morphology-related qualifiers where appropriate to the selected organ.

#### Data analysis and visualization

A significant motivation for the generation of MPHASYS is the ability to analyze and share data. In addition to the capabilities of the data entry tool for analysis of animal data, the framework was used to create web-based analysis tools to present and analyze data via a web-browser. Animal data from multiple collection points can be entered into a single server running the web application and the data can be compared across studies. A user can define selection criteria for analyzing animal data and visualize the results within the application. Using these criteria, the system can be used to visualize clinical variables for individual animals as a function of age or life span. Charts are interactive and can be used to gain specific information about individual animals via tooltips.

#### Image data

The framework has programmatic tools for uploading, annotation and display of histopathological images. Images that are captured by histopathologists at the microscope can be loaded into MPHASYS through a the application, where they are associated with the individual animal and the pathological finding (topography, morphology and qualifiers). Images are presented through the web interface and automatically annotated with codes from the ontology.

#### Access modes

Finally, the application can be configured to either operate in a single- or multi-user environment. The single user mode (the default for the client application) utilizes a local embedded HSQLDB database while the multi-user mode accesses a stand-alone database server (*e.g*. PostgreSQL). Use of a common database server allows for multi-user access to common animal data. Studies can be exported and shared as *MouseML *documents, allowing investigators a method of integrating published data into their own analyses. When documents are shared and loaded, the application not only validates the data to ensure it is of the proper type, but will keep an audit trail of any changes made to existing data. For additional information on configuration of MPHASYS access modes, please see the online documentation [1].

### Example: collection and analysis of wild-type and Xpd^TTD ^mouse data

The mouse data used to illustrate the features and functions of MPHASYS were taken from a published study of aging-related pathologies in Xpd^*TTD *^mice [24] and unpublished data. Xpd^*TTD *^mice harbor a hypomorphic mutation in the Ercc2 gene that mimics the human disease trichothiodystrophy. These animals present reduced life-span and enhanced age-related pathology. Data related to the wild-type and Xpd^*TTD *^animals were entered using the client application (pathology data) and spreadsheet bulk-loading tools based on the framework (weight data). Data collection through the forms-based client application offers a significant advantage over the more traditional spreadsheet-based data collection, in that there is a much lower chance for data corruption by accidental deletion/alteration or well-intentioned but deleterious reformatting by spreadsheet software.

**Figure 5:**
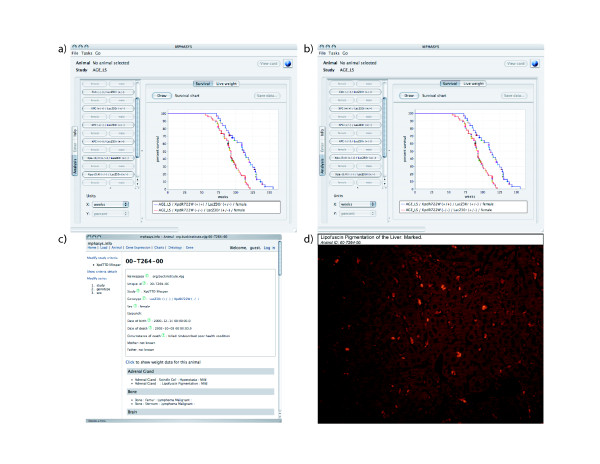
**Analysis of animal data using the MPHASYS applications**. By selecting criteria for analysis, the user can generate graphical reports of life-span animal data. a) Survival analysis of Xpd^*TTD *^and wild-type females using the client application. b) Comparative weights of Xpd^*TTD *^and wild-type females using the client application. c) Individual animal histopathology report using the web-application. d) Image of representative increased lipofuscin pigmentation in Xpd^*TTD *^mice.

Using the web application or the data entry tool, selection criteria can be defined to select subsets of animals. Selecting female Xpd^*TTD *^animals and their wild-type counterparts allows for analysis of their properties in the analysis modules by choosing genotypes and sex (in the client application) or by building a logical expression (web application). The charting tools demonstrate significant Xpd^*TTD*^-related changes in life-span and weight for the animals selected in this example (Figure 5) as reported in [24]. Inspection of the weight charts indicates that a precipitous drop in weight is associated with death in wild-type animals, but generally not in the already underweight Xpd^*TTD *^animals. The charts also indicate that overweight animals tend to die at a younger age. The data visualization tools also allow direct inspection of individual animals for histopathological characteristics that may offer clues to their premature demise. For example, clicking on an animal in the web application takes you to the individual animal's record where histopathology can be inspected (Figure 5c). Choosing abnormally heavy animals for inspection would indicate, for example, that animals with high body weight are not associated with neoplastic pathologies. This report also presents an animal's weight in the context of its own genotype and sex for comparison. Images that are associated with pathologies can be inspected for comparison. For example, UV micrographs of lipofuscin pigmentation of the liver were captured for Xpd^*TTD *^and wild-type animals, demonstrating an Xpd^*TTD*^-related increase in the deposition of the aging-related pigment lipofuscin [32] (Figure 5d).

### Future design goals

The extensible core of the MPHASYS framework provides a convenient mechanism for integration of biologically meaningful data. For example, molecular data, *e.g. *gene expression data, can be linked to the organs of individual animals to enable correlation of gene expression with specific pathologies. With a complete picture of how within an individual organism clinical, pathological and molecular variables interrelate, a broader, more extensible analysis will be attainable. MPHASYS will provide direct access to such primary animal data as well as new tools for data visualization, data mining and hypothesis generation.

For example, patterns of individual pathology can be mined using unsupervised classification in order to find age, genotype, and treatment related patterns of histopathology. These patterns can then be correlated with patterns of gene expression to propose questions about molecular processes and their involvement in aging and responses to DNA damage.

## Conclusion

We have developed a new computational platform that allows the collection of high-quality data from individual animals including their specific patterns of pathology in an ontology mediated protocol. This platform serves as the basis for tools for data capture and validation as well as analysis. MPHASYS utilizes a code-based ontology for collection of data as a tool for rapid and accurate collection of pathology data. As the tools for working with ontologies evolve and are incorporated into intelligent systems (i.e. systems that make logical assertions based on an upper ontology like the Suggested Upper Merged Ontology (SUMO) or OpenCyc), data collected using MPHASYS will have adequate descriptions to be utilized. As more data from studies on these and other animals are obtained and used to populate MPHASYS, it will be possible to conduct *in silico *studies, test hypotheses and design new ones. The future integration of different types of data (gene expression profiles) on individual animals will generate more complete individual animal phenomic signatures that can be compared across experiments in a validated well defined format that can be shared with other investigators. Comparisons can be made with animal phenomes from other studies, without the need to re-integrate legacy data into a new analysis. MPHASYS provides tools for data entry, analysis and dissemination of animal data. The data model is designed in such a way that other forms of data can easily be associated with individual animals.

## Availability and requirements

The MPHASYS application framework and client application are freely available and distributed under the GNU LGPL license [1]. It has been developed in the Java programming language and requires a virtual machine of version 1.4.2 or higher.

**Project name: **MPHASYS

**Project home page: **

**Operating system(s): **Platform independent

**Programming language: **Java

**Other requirements: **Java 1.4.2 or higher

**License: **GNU LGPLv2

**Any restrictions to use by non-academics: **none

## Authors' contributions

JV conceived and supervised the study, and obtained the funding for it. Design and modeling by RBC, ISM and PHML. Architecture and implementation by RBC. Animal data, analysis and additional terms by RBB and HvS. All authors read and approved the final version of this manuscript.
